# Robotic Compartment-Based Radical Surgery in Early-Stage Cervical Cancer

**DOI:** 10.1155/2016/4616343

**Published:** 2016-04-18

**Authors:** Tayfun Toptas, Aysel Uysal, Isin Ureyen, Onur Erol, Tayup Simsek

**Affiliations:** ^1^Department of Gynecologic Oncological Surgery, Antalya Research and Training Hospital, 07070 Antalya, Turkey; ^2^Department of Obstetrics and Gynecology, Antalya Research and Training Hospital, 07070 Antalya, Turkey; ^3^Department of Gynecologic Oncological Surgery, Akdeniz University School of Medicine, 07070 Antalya, Turkey

## Abstract

A radical hysterectomy with pelvic lymphadenectomy is the recommended treatment option in patients with early-stage cervical cancer. Although various classifications were developed in order to define the resection margins of this operation, no clear standardization could be achieved both in the nomenclature and in the extent of the surgery. Total mesometrial resection (TMMR) is a novel procedure which aims to remove all components of the compartment formed by Müllerian duct in which female reproductive organs develop. TMMR differs from the conventional radical hysterectomy techniques in that its surgical philosophy, terminology, and partly resection borders are different. In this paper, a TMMR with therapeutic pelvic lymphadenectomy operation that we performed for the first time with robot-assisted laparoscopic (robotic) approach in an early-stage cervical cancer patient was presented. This procedure has already been described in open surgery by Michael Höckel and translated to the robotic surgery by Rainer Kimmig. Our report is the second paper, to our knowledge, to present the initial experience regarding robotic TMMR in the English literature.

## 1. Introduction

Cervical cancer is a global health problem for women. According to the World Health Organization, the number of newly diagnosed cases worldwide is 528,000 in 2012, and the number of deaths caused by the disease is 266,000 [1]. In developed countries, a clear decrease was achieved in incidence and mortality with the widespread use of screening programs; however, it continues to be one of the leading causes of cancer deaths in developing countries [2].

Cervical cancer can be treated primarily with surgery or radiotherapy in early stages. Surgery is typically preferred in small lesions such as stages IA and IB_1_ and some selected stage IIA_1_ [3]. While extrafascial hysterectomy is sufficient in tumors with a depth of invasion ≤ 3 mm and no lymphovascular space involvement (LVSI), radical hysterectomy with pelvic lymphadenectomy is recommended in the remaining early-stage tumors [4].

A radical hysterectomy operation was first described by Wertheim more than a century ago [5]. Up to now, various classifications were developed in order to precisely define the resection margins of this operation [6, 7]. However, no clear standardization could be achieved both in the nomenclature and in the extent of the surgery. In 2003, Höckel et al. [8] put forth the ontogenetic compartment theory with regard to this subject. According to this theory, until late in disease progression, the local-regional spread of a tumor is confined to the compartment derived from a common primordium that the relevant organ originated from in its embryonic development. Thus, the removal of all components of the relevant compartment and its lymphatic drainage routes in tumors that tend to spread primarily locally may ensure the local control of the disease without adjuvant therapy [9]. In line with their theory, Höckel et al. named the removal of the compartment formed by Müllerian duct in which female reproductive organs develop as “total mesometrial resection (TMMR)” [8].

In this paper, a TMMR with therapeutic pelvic lymphadenectomy operation that we performed for the first time with robot-assisted laparoscopic (robotic) approach in a stage IA_2_ cervical cancer patient was presented with the technical details.

## 2. Case Presentation

This work was conducted in accordance with the Declaration of Helsinki and with approval from the Institutional Ethics Committee. Written informed consent was obtained from the patient.

A 34-year-old, gravidity one, parity one, woman was admitted to our clinic with a cotesting result of high-grade cervical intraepithelial lesion (HSIL) with human papilloma virus type 16 positivity. She was 48 kg in weight with a body mass index of 21 kg/m^2^. Colposcopically directed biopsy was consistent with cervical intraepithelial neoplasia-3 (CIN_3_). She underwent cold knife conization. Histopathological examination revealed a nonkeratinizing squamous cell carcinoma with a stromal invasion 4 mm in depth and 6 mm in horizontal spread.

A robotic TMMR with therapeutic pelvic lymphadenectomy procedure was planned following the detailed information of the patient about the treatment options. The patient was also clearly informed that there is no gold standard surgical technique in the treatment of cervical cancer and the radicality of the surgery is still debatable, especially for such an early-stage disease.

### 2.1. Authors' Learning and Implementation Process for Robotic TMMR

In 2014, all authors attended three-day simulation-based training curricula designed to improve robotic surgical skills at the Center of Advanced Simulation and Education (CASE), Acıbadem University, in Istanbul, and successfully completed both the theoretical and practical tests. After the first 30 cases of procedures for benign gynecologic pathologies and 10 cases of staging procedures for early-stage gynecologic cancers, we felt more confident in our ability to implement robotic surgery and decided to perform a robotic TMMR with therapeutic lymphadenectomy. This procedure has already been described in open surgery by Höckel et al. [8] and systematically translated to the robotic surgery by Kimmig et al. [10]. We intensively studied the scientific papers of both authors regarding the surgical technique of this procedure [8–11] and benefited much from the high-definition (HD) educational surgical video modules that are accessible from the website of the European Society of Gynaecological Oncology (ESGO e-academy) [12, 13]. We also participated in the 7th annual meeting of the Society of European Robotic Gynecologic Surgery (SERGS), which was held in June 2015, in Istanbul. In this meeting, we found an opportunity to watch the live surgery of robotic TMMR by Kimmig.

### 2.2. Surgical Technique

As the distance between the umbilicus and symphysis pubis of the patient was short, the abdomen was entered 3 cm above the umbilicus in the midline in a way that the distance to the target organ (uterus) was ~20 cm. Pneumoperitoneum was created using a Veress needle, and the maximum pressure was set at 14 mmHg. An 8 mm robotic trocar was inserted from the same incision. The robotic camera was passed through this trocar, and two other robotic trocars were then inserted 8 cm lateral to both sides of the camera trocar under laparoscopic vision. An additional fourth robotic trocar was inserted 6 cm lateral to the third trocar on the right side, and a 12 mm assistant port was placed at the left upper quadrant between the camera trocar and the left robotic trocar.

The patient-side cart of the robotic surgical system (da Vinci® XI, Intuitive Surgical Inc., Sunnyvale, CA) was approached to the patient from the right side and docked to the patient. The monopolar scissors were inserted through the right robotic trocar; Maryland bipolar forceps, through the left robotic trocar; and ProGrasp*™* forceps, through the fourth robotic trocar.

The operation was carried out in three stages: first, bilateral ovarian transposition was applied; therapeutic pelvic lymphadenectomy was performed in the second stage; and Müllerian compartment resection was performed in the third stage.

**Figure 1:**
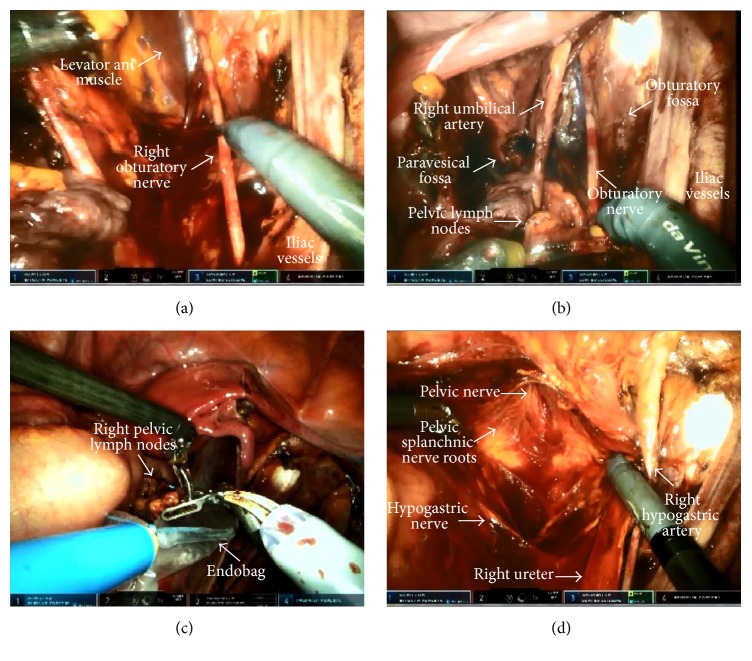
Therapeutic pelvic lymphadenectomy (right side). (a, b) The view of the right pelvic side wall after lymph node dissection. (c) Placing the dissected lymph nodes into Endobag*™*. (d) The view of the hypogastric nerve, pelvic splanchnic nerve roots, and pelvic nerve following the dissection of the medial pararectal fossa.

Therapeutic pelvic lymphadenectomy was performed in accordance with the technique described by Kimmig et al. [11] including en bloc resection of the local (intercalated lymph nodes (LNs) in the vascular mesometrium) and the regional lymphatic systems (paravisceral, external iliac, hypogastric, common iliac, obturatory, and presacral LNs) (Figure 1). The specimens were placed into endoscopic surgical bags (Endobag) and left in the abdomen so as to remove them from the vagina at the end of the operation. Afterwards, the ureter was entirely separated along with the mesoureter from the medial leaf of the peritoneum to the ureteric tunnel. The medial pararectal fossa (Okabayashi space) was dissected until the hypogastric nerve, pelvic splanchnic nerve roots, and the pelvic nerve were identified. The vascular mesometrium was revealed by dissecting the lateral pararectal fossa (Latzko space) in a similar depth.

**Figure 2:**
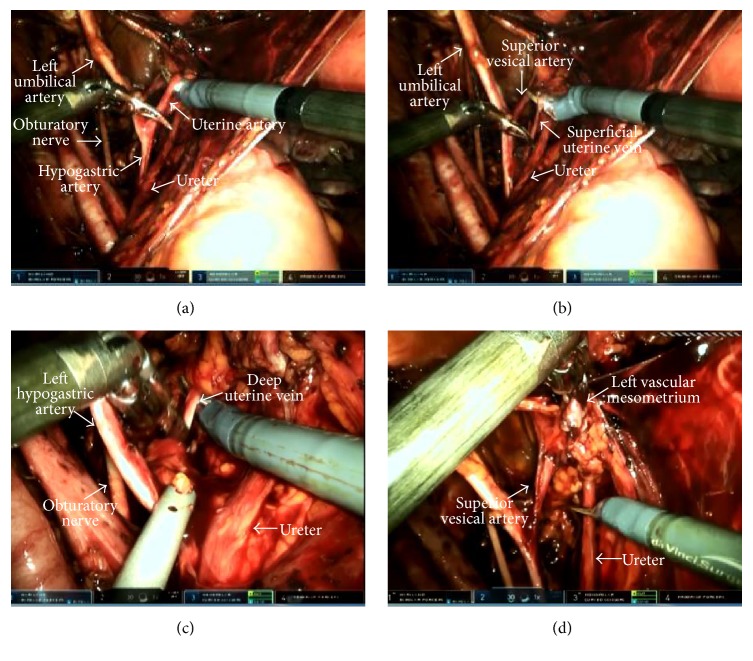
Resection of the lateral part of the vascular mesometrium (left side). (a) Resection of the uterine artery. (b) Resection of the superficial uterine vein. (c) Resection of the deep uterine vein. (d) The view of the resected mesometrial bundle.

**Figure 3:**
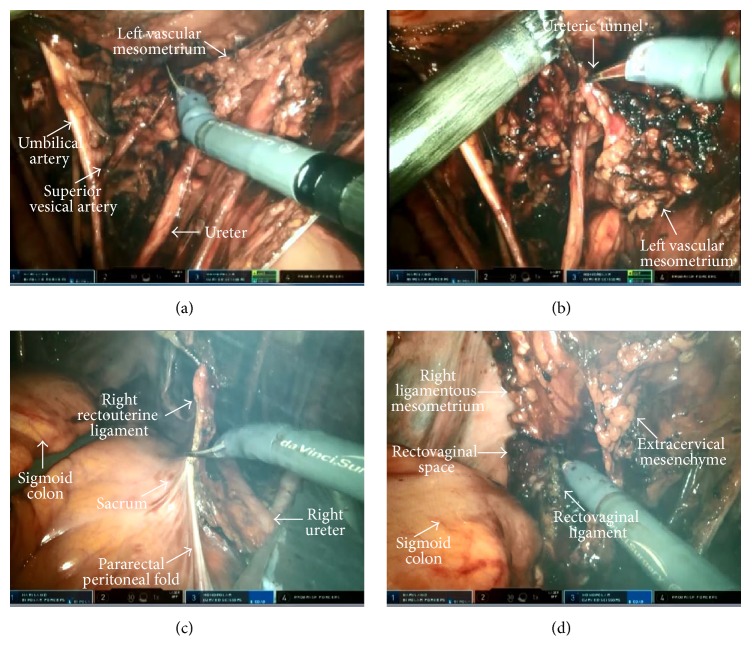
((a), (b)) Resection of the anterior part of the vascular mesometrium (left side). ((c), (d)) Resection of the ligamentous mesometrium (right side).

The resection of the Müllerian compartment was completed in two steps being the resection of the vascular mesometrium and ligamentous mesometrium. In the resection of vascular mesometrium, first, the lateral connections (lateral parametrium) including uterine artery, superficial uterine vein, parametrial LNs, and the deep uterine vein were cauterized and cut at their origin from the hypogastric vessels, in the vertical plane, respectively (Figure 2). The lateral mesometrial bundle that was resected was elevated towards the ureter using ProGrasp forceps. Then, the vesicovaginal space was dissected by 3-4 cm in order to reveal the upper two-thirds of the vagina that constitutes the lowest part of the Müllerian compartment. This was aimed to identify the border of the vesical and Müllerian compartments and thus to facilitate the dissection of the vesicovaginal arterial anastomoses in the anterior part of vascular mesometrium and the preservation of the ureteral branches of the bladder mesentery. Subsequently, the anterior connections (ventral parametrium) of the vascular mesometrium were incised in such a way to ensure that the lateral borders were superior vesical artery and the ureterovesical junction. Thus, the ureteric tunnel was opened, the ureter was separated from the Müllerian compartment, and the resection of the vascular mesometrium was completed (Figures 3(a) and 3(b)).

Resection of the ligamentous mesometrium is presented in Figures 3(c) and 3(d). First, the rectovaginal space was opened and the rectum was mobilized from the posterior vaginal wall. Then, the pararectal peritoneal folds were incised from the level of common iliac artery to the level of medial aspect of the ligamentous mesometrium which consists of rectouterine and rectovaginal ligaments. The incision was continued downwards to the previously dissected rectovaginal plane following the transection of rectouterine and rectovaginal ligaments at their perirectal attachments, along with the lymphatics draining into the prespinal and preischiadic nodes of the paravisceral LN compartment.

**Figure 4:**
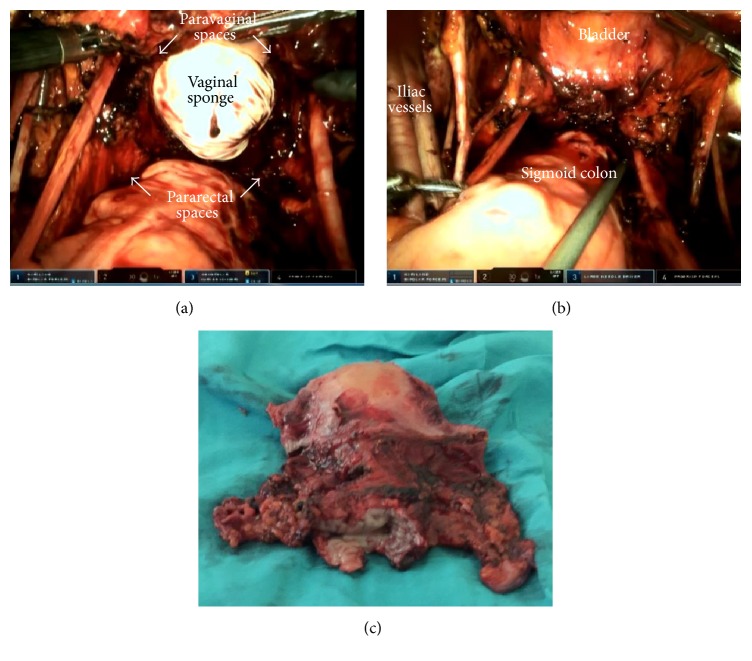
((a), (b)) Final view of the pelvis after total mesometrial resection. (c) Surgical specimen.

Following the complete mobilization of the vascular and ligamentous parts of the mesometrium, the upper vagina was resected circumferentially with at least 1 cm of clear surgical margins confirmed by histology. In contrast to other parts of the Müllerian compartment, the vaginal part was not removed completely to avoid vaginal shortening and dysfunction. A uterine manipulator was not used in any step of the procedure. Instead, a sponge stick that was covered with a glove was applied in case of need. All specimens and surgical bags were retrieved vaginally. Finally, the vagina was closed with a running barbed suture using V-Loc*™* 90 absorbable wound closure device (Covidien, Mansfield, MA). The view of the pelvis after TMMR is shown in Figure 4.

### 2.3. Perioperative Findings

The operating times were as follows: docking time, 20 minutes; console time, 300 minutes; anesthesia time, 340 minutes; and theatre time, 360 minutes. No complication was observed intraoperatively. In total, 200 mL of bleeding occurred. She was fully mobilized and a liquid diet was started on postoperative day one. On the second day, a regular diet was started and the urinary catheter was removed. The amount of residual urine volume that was measured by recatheterization on the third day was 50 mL. She had an uneventful postoperative course and was discharged home on postoperative day five. The final histopathological examination revealed a residual tumor of 1 mm in size with negative surgical margins, parametria, and LNs (0/28). She did not receive adjuvant therapy and has been followed up for 9 months without any clinical problem.

## 3. Discussion

Although more than 20 years passed after the description of laparoscopic approach for the surgical treatment of cervical cancer by Nezhat et al. [14], it has not been widespread due to its long learning curve, technical limitations, ergonomic problems, and the necessity for advanced laparoscopic skills. With the introduction of surgical robotics into clinical practice in the mid-2000s, many limitations of the conventional laparoscopy have been eliminated, and this led to the rapid popularity of this new technology.

In 2006, Sert and Abeler [15] first reported the robotic technique for radical hysterectomy. Following the initial reports, several studies have compared the surgical outcomes between robotic, conventional laparoscopic, and open techniques and indicated the safety and feasibility of robotic approach [16–18].

In a case-control study comparing robotic (*n* = 51) versus open (*n* = 49) approach, Boggess et al. [16] demonstrated that the robotic approach was associated with shorter hospital stay (1 versus 3.2 days) and lower blood loss (96.5 ± 85.8 versus 416.8 ± 188.1 mL). Though not statistically significant, the complication rate was also lower in the robotic group (7.8% versus 16.3%). The authors noted that the operative time significantly decreased after the first 12 cases (243.4 versus 193.2 minutes), which may indicate the learning curve of this technique. Magrina et al. [17] compared the perioperative outcomes of patients undergoing radical hysterectomy by robotics (*n* = 27), laparoscopy (*n* = 31), and laparotomy (*n* = 35) and reported that operating times for robotics (189.6 min) and laparotomy (166.8 min) were similar and significantly shorter as compared to laparoscopy (220.4 min). Blood loss and length of hospital stay were similar for robotics and laparoscopy and significantly reduced as compared to laparotomy. The extent of parametrial tissue resection was similar for each group. In a matched-case comparative study assessing the disease outcomes after robotic (*n* = 23) and conventional laparoscopic (*n* = 69) techniques, Kim et al. [18] reported similar overall 3-year recurrence-free survival rates between the two techniques (91.3% for robotic group versus 89.9% for laparoscopy group; *p* = 0.778).

The surgical technique of the TMMR operation is practically similar to that of the Querleu-Morrow Type C_1_ radical hysterectomy [7]; however, it differs from the other one in that its surgical philosophy, terminology, and partly resection borders are different. The philosophy of the TMMR relies on the ontogenetic theory of locoregional tumor spread [19], which provides an essential oncological rationale for our surgical practice. However, the historical Querleu-Morrow classification describes only what should the resection margins be in radical hysterectomies, regardless of any oncological point of view. The resection borders of the vascular mesometria (lateral and ventral parametria) are identical in each technique both in transverse and in vertical planes, but, in the resection of ligamentous mesometria (posterior parametrium), TMMR includes the resection of rectovaginal ligament, extracervical mesenchyme, and proximal vagina up to the lower limit of the rectovaginal ligament in addition to the resection of rectouterine ligament [8]. Furthermore, a complete resection of the draining lymphatic compartments, the so-called “therapeutic lymphadenectomy,” is one of the essential parts of the TMMR. By contrast, with different types of not well-defined “staging lymphadenectomies,” the therapeutic lymphadenectomy aims at not only staging of the disease but also removing of all LNs of certain lymphatic basins and the intercalated LNs at risk for involvement [11].

The robotic technique for TMMR was first described by Kimmig et al. in 2013 [10]. In a case series consisting of 26 consecutive patients with stage IA-IIB cervical cancer, the authors noted that there was no need for conversion to open surgery. No intraoperative complications were reported. Six patients (23%) experienced postoperative complication; one had vaginal cuff dehiscence, two developed infected lymphocyst, two had wound infection, and relaparoscopy was performed due to bleeding in one patient. Of the patients, 22% had LN involvement at final histopathology. During the mean follow-up period of 18 months, one patient developed distant metastasis, but no local-regional recurrence was reported.

Our report is the second paper, to our knowledge, to present the initial experience regarding robotic TMMR in the English literature. We demonstrated how modern techniques including robotic surgery, HD video documentation, and educational/scientific publications and presentations may enable a surgical team to successfully adopt a certain concept or innovative surgical procedure into their clinical practice. Although we did not have any TMMR experience, the surgery was satisfactory enough both for the patient and for the surgical team. All the anatomical landmarks were clearly presented. No perioperative complication was observed. The patient could be discharged home and returned to her daily activities in the early period without any problems. Although our console time of 300 minutes was considerably long, this would improve with time as we get beyond the learning curve of this new technique.

The concept of compartment-based radical surgery in cervical cancer aims at resection of a topographically defined anatomical territory for achieving local tumor control without adjuvant therapy [9]. Thus, in this concept, there is no need for transposition of the ovaries. However, we preferred to perform this procedure in our patient due to the fact that this kind of management strategy is quite new for many radiation and medical oncologists in our country, so that, in case of presence of any classical risk factors for radiotherapy in final pathology, it might lead to conflicts between the members of multidisciplinary tumor board in decision-making for adjuvant therapy.

Radical hysterectomy with pelvic lymphadenectomy is often associated with varying degrees of bladder dysfunction caused by surgical trauma to autonomic nervous system. Over the past 20 years, attempts to prevent urinary morbidity through the preservation of pelvic autonomic nerves have led to the development of nerve-sparing concept in the treatment of cervical cancer. A recent systematic meta-analysis comparing the efficacy and safety of conventional and nerve-sparing radical hysterectomies indicated that nerve-sparing technique was significantly associated with lower urinary morbidity and faster recovery of pelvic organ functions, while the two techniques were similar in extent of resection and survival rates [20]. However, the interpretation of these results is somewhat problematic since both procedures are not well standardized and may differ considerably among individuals. TMMR in itself is a functional and conservative radical surgery which aims at resecting the target morphogenetic units while keeping pelvic-tissue trauma at a minimum. In contrast to historical radical hysterectomies, the bladder mesenteries, mesorectum, and autonomic nervous system are exposed and preserved [9]. Robotic approach in TMMR may facilitate the identification and preservation of nerve fibers and other anatomical structures by providing three-dimensional vision, by wrist articulation, and by negating tremors. In our patient, no immediate or late postoperative urinary symptoms were observed despite the early removal of urinary catheter. However, it is evident that the available literature is in its infancy and the role of robotic surgery and of nerve-sparing techniques in prevention of urinary morbidity remains a subject of further trials.

In conclusion, robotic TMMR is a minimally invasive treatment option for patients with early-stage cervical cancer that can be easily and successfully adopted into clinical practice. Further comparative clinical trials with sufficient follow-up data are needed to validate the long-term oncological and functional outcomes of this technique.
